# Historical factors shaped species diversity and composition of *Salix* in eastern Asia

**DOI:** 10.1038/srep42038

**Published:** 2017-02-08

**Authors:** Qinggang Wang, Xiangyan Su, Nawal Shrestha, Yunpeng Liu, Siyang Wang, Xiaoting Xu, Zhiheng Wang

**Affiliations:** 1 Department of Ecology, College of Urban and Environmental Sciences, and Key Laboratory of Earth Surface Processes of Ministry of Education, Peking University, Beijing 100871, China

## Abstract

Ambient energy, niche conservatism, historical climate stability and habitat heterogeneity hypothesis have been proposed to explain the broad-scale species diversity patterns and species compositions, while their relative importance have been controversial. Here, we assessed the relative contributions of contemporary climate, historical climate changes and habitat heterogeneity in shaping *Salix* species diversity and species composition in whole eastern Asia as well as mountains and lowlands using linear regressions and distance-based redundancy analyses, respectively. *Salix* diversity was negatively related with mean annual temperature. Habitat heterogeneity was more important than contemporary climate in shaping *Salix* diversity patterns, and their relative contributions were different in mountains and lowlands. In contrast, the species composition was strongly influenced by contemporary climate and historical climate change than habitat heterogeneity, and their relative contributions were nearly the same both in mountains and lowlands. Our findings supported niche conservatism and habitat heterogeneity hypotheses, but did not support ambient energy and historical climate stability hypotheses. The diversity pattern and species composition of *Salix* could not be well-explained by any single hypothesis tested, suggesting that other factors such as disturbance history and diversification rate may be also important in shaping the diversity pattern and composition of *Salix* species.

Understanding the macro-scale species diversity patterns and the underlying mechanisms is one of the central challenges in ecology and biogeography^1^,^2^. In spite of theoretical advancement and a large number of studies in the last a few decades, many controversies still remain in the current literature. In order to determine the relative importance of mechanisms of the species diversity patterns, it is equally important to explore the species composition across large spatial scales in addition to exploring species diversity (i.e. species number).

Species diversity generally decreases with latitude and this pattern has been largely shaped by contemporary climate. One of the most widely studied hypotheses is the ambient energy hypothesis, which states that ambient energy imposes environmental carrying capacity on the number of individuals and consequently on species diversity^3^,^4^. It predicts that species diversity is positively correlated with the environmental temperature. This hypothesis has been tested widely and strong positive species diversity-temperature relationships have been reported for different taxonomic groups (e.g. birds^5^ and woody plants^6^).

However, recent studies have shown that species diversity-climate (including diversity-energy and diversity-water) relationships varies across clades with different evolutionary history due to niche conservatism^7^, and hence supported niche conservatism hypothesis^8^. Particularly, niche conservatism hypothesis regards that the latitudinal gradients of species diversity is rooted in the evolution of particular clades. Specifically, this hypothesis predicts that species diversity for a given clade will be low in regions where the climate conditions deflect from the clade’s ancestral niche, because species have difficulties to evolve adaptions to new climatic niches^8^,^9^,^10^. According to niche conservatism hypothesis, species originated in tropical regions tend to show strong positive diversity-temperature relationship while clades originated in temperate regions show negative diversity-temperature relationship^11^,^12^,^13^. However, it still remains controversial whether contemporary climate drives species diversity through the control of ambient energy or through niche conservatism.

In addition to the controversy on the relative contribution of contemporary climate vs. deep-time evolution, many authors proposed that climate changes since the Last Glacial Maximum (LGM) (ca. 21,000 years before present) also influence species diversity patterns^14^,^15^. Therefore, the place experiencing the most severe climate changes have less species (i.e. the history climate stability hypothesis)^16^. Studies on the distribution of European trees found that climate changes since the LGM have led to the confinement of species’ distribution ranges to the Mediterranean regions^17^,^18^, and many species still do not inhabit the climatically suitable regions in northern Europe due to dispersal limitation^19^. In addition to species diversity, climate changes also significantly influence species composition^20^,^21^. Understanding how species composition responds to climate changes may improve our ability to predict novel communities under future climate change scenarios^21^. However, most of previous studies have focused on the effects of historical climate changes on species diversity, while the effects of historical climate changes on species composition remain poorly understood.

Habitat heterogeneity hypothesis predicts that topographic heterogeneity (e.g. elevation range and slope) contribute to the variation of species diversity^22^,^23^. Previous study on bird richness in South America showed that the model including topography relief exhibited the highest explanatory power^24^. Habitat heterogeneity may influence species diversity through the increase of available ecological niches which support more species^3^,^25^ and enhanced diversification due to the effects of meso-scale climate gradients^26^. However, the relative importance of habitat heterogeneity and climate on species diversity and composition is controversial.

Compared with surrounding lowlands, mountains have much higher topographical heterogeneity and cooler climate (especially cooler summer) because with every 100 meters rise in the altitude, the atmospheric temperature decreases by about 0.5 to 0.6 °C. In addition, mountains experience less human disturbance than the lowlands^27^ and could act as refuges both in the past and future. Mountains therefore have been the focus of conservation efforts^27^,^28^. Comparison of the determinants of species diversity and species composition in mountains *vs*. lowlands may enhance for nature conservation efforts in global mountains.

*Salix* is one of the main groups of woody plants in the North Temperate Zone^29^,^30^,^31^,^32^. The genus *Salix* consists of about 450–520 species of shrubs and trees^31^. The vast majority of *Salix* species are important components in riparian zone of desert, plain and mountains. They are also dominant species in alpine shrubland and cushion vegetation. Fossil evidence suggests that *Salix* originated in late Cretaceous in the temperate region of the northern Hemisphere^33^,^34^,^35^. With regard to their temperate origination, following niche conservatism hypothesis, we expect that the energy related variables would be negatively correlated with *Salix* diversity. Moreover, many *Salix* species are found in mountain slopes of eastern Asia^32^. Therefore, in addition to niche conservatism, we predict that habitat heterogeneity plays an important role in shaping *Salix* diversity pattern in eastern Asia. However, the quantitative assessment of the pattern of *Salix* diversity in eastern Asia and its relationships with climate and habitat heterogeneity remained largely elusive.

In this study, using the distribution maps of 313 *Salix* species in eastern Asia, we aimed to (1) assess the patterns of *Salix* species diversity and composition, (2) explore the relationship between species diversity/composition and environment (i.e. contemporary climate, historical climate changes and habitat heterogeneity). In our analysis, the following questions were addressed: (1) which hypothesis (i.e. ambient energy, niche conservatism, historical climate stability and habitat heterogeneity) dominates the patterns of *Salix* diversity? (2) What is the relative contribution of contemporary climate, historical climate changes and topographic heterogeneity in shaping *Salix* species composition? (3) Do the answers to these two questions differ between mountains and lowlands?

## Materials and Methods

### *Salix* species distribution data

In this study, the species distributions of *Salix* in eastern Asia were compiled from various resources (see below). Here, the eastern Asia referred to China, Mongolia, Asian part of Russia and central Asia. The distributions of *Salix* in China were obtained from the *Atlas of Woody Plants in China*^36^ and National Specimen Information Infrastructure (NSII) (http://www.nsii.org.cn/). *Salix* distribution in Mongolia was obtained from *Virtual Guide to the Flora of Mongolia Plant Database as Practice Approach* (http://greif.uni-greifswald.de/floragreif/), and the data of the Asian part of Russia and central Asia were from *Woody Plants of the Asian Part of Russia* and *Flora of Soviet Union* (I, II and III). In total, there are 17,189 county-level distribution records in *Atlas of Woody Plants in China* and 7,181 georeferenced specimen records for *Salix* distribution in China and 284 digitized distribution maps for those in Mongolia, Asian part of Russia and central Asia. To eliminate the influence of area on the estimation of species diversity, the species distribution data were gridded into an equal-area grid with grain size of 100 × 100 km. We excluded grid cells with land area located on the borders or along coasts that have land area less than 2500 km^2^. As a result, 2685 grid cells were finally included in the following analyses. We also conducted all the analyses using at 50 × 50 km grid cells, and all results are consistent. Therefore, we reported the results based on the grid of 100 × 100 km in the manuscript, and those based on the grid of 50 × 50 km were in Supplementary Materials (Tables S1–S2, Figs S1–S3).

### Environmental data

We obtained contemporary climate and GLM climate data with the resolution of 30 arc-second from the WorldClim website (www.worldclim.org/)^37^. The contemporary climate data include mean monthly temperature and precipitation, mean annual temperature (MAT), mean temperature of coldest quarter (MTCQ), mean temperature of driest quarter (MTDQ), mean annual precipitation (MAP) and mean precipitation of driest quarter (MPDQ). Then using the mean month temperature and precipitation, we estimated annual rainfall (RAIN) as the total precipitation of the months with mean monthly temperature >0 °C^38^. We also obtained the annual potential evapotranspiration (PET) and actual annual evapotranspiration (AET) from CGIAR-CSI Global PET database (www.cgiar-csi.org/data/global-aridity-and-pet-database)^39^ and CGIAR-CSI Global Soil-Water database (http://www.cgiar-csi.org/data/global-high-resolution-soil-water-balance)^40^. The LGM climate data include the mean annual temperature and mean annual precipitation during LGM which calculated based on MIROC-ESM model. To reflect the historical climate changes, we calculated the anomaly of mean annual temperature and mean annual precipitation (MAT anomaly and MAP anomaly) as contemporary mean annual temperature and contemporary mean annual precipitation minus those at the Last Glacial Maximum respectively (Table S3).

We used elevational range and mean slope of each grid cell to represent habitat heterogeneity using the Digital Elevation Model derived from Global 30 Arc-second Elevation (GTOPO30) of U.S. Geological Survey (https://lta.cr.usgs.gov/GTOPO30)^41^. Elevational range was estimated as the difference between the maximum and minimum elevations within each 100 × 100 km grid cell. Slope was calculated with the Slope Tool in the spatial analysis module of ArcGIS 10.0 (ESRI, Inc., Redlands, California, USA). We first calculated the slopes within the study region at the spatial resolution of 1 × 1 km and then took the average slope of all 1 × 1 km grid cells within each 100 × 100 km grid cell.

### Mountains *vs*. lowlands

**Figure 1:**
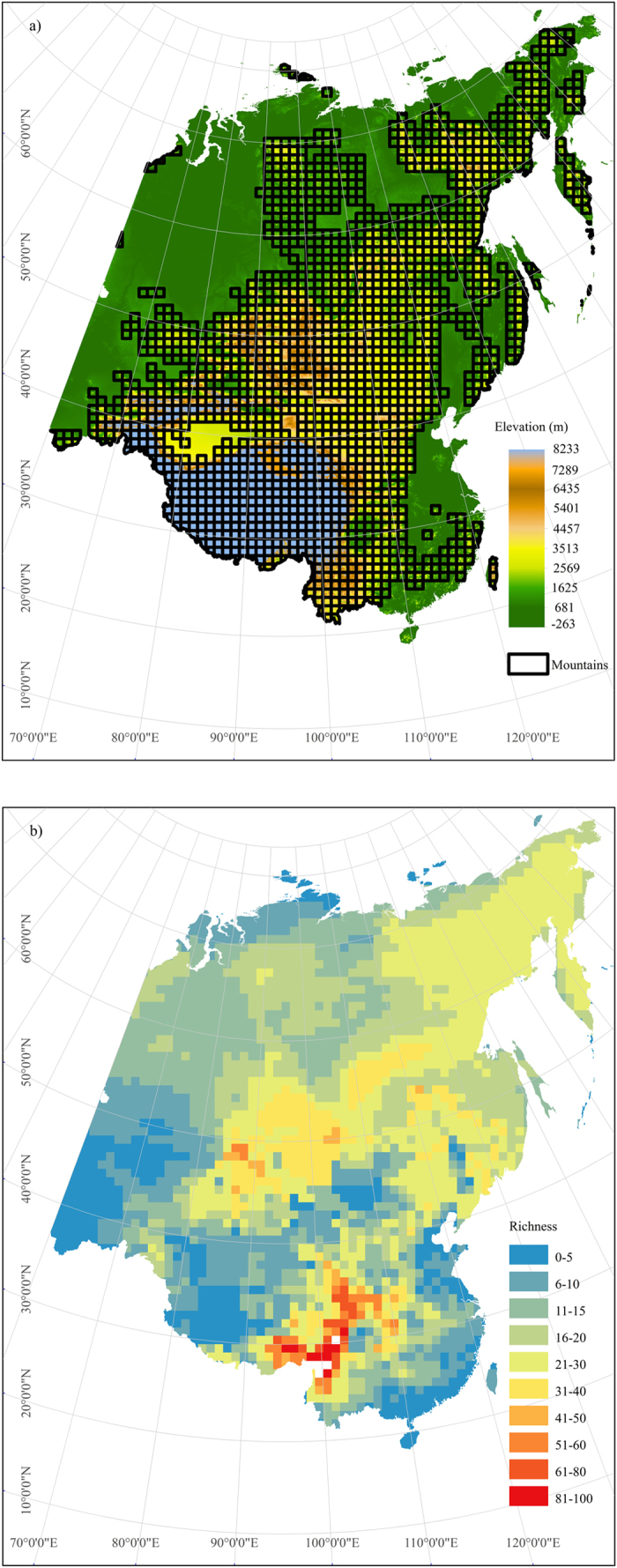
The distribution pattern of *Salix* species diversity in eastern Asia estimated in an equal-area grid of 100 × 100 km and the topography map of eastern Asia. (**a**) The topography map; b) the distribution pattern of *Salix* diversity. The maps were created in ArcGIS 10 (http://www.esri.com/software/arcgis). The topography map in the figures was generated based on Global 30 Arc-second Elevation (GTOPO30) of U.S. Geological Survey (https://lta.cr.usgs.gov/GTOPO30).

We defined mountains following the criterion of United Nations Environmental Program/World Conservation Monitoring Centre^42^, which includes the following criteria. Particularly, a grid cell was counted as mountain if 1) its mean elevation is >2500 m; or 2) its mean elevation is 1500–2500 m and mean slope is >2°; or 3) its mean elevation is 300–1500 m and elevational range is >300 m. Following this criteria, 62.0% of 100 × 100 km grid cells were defined as mountains (Fig. 1a).

### Data analyses

#### Regressions for species diversity

General linear models were used to assess the explanatory power of each climate and habitat variable for *Salix* species diversity pattern in the whole study region, mountains and lowlands, respectively. Species diversity are square-root transformed to improve normality following previous study^43^. The spatial autocorrelation in species diversity could inflate type I error, and subsequently the significance levels of the statistical tests for regression models. Therefore, we used modified t-test to test the significance of all regression coefficients^44^.

#### Distance-based redundancy analysis for species composition

Species composition for *Salix* in the whole study region, mountains and lowlands were analyzed separately using distance-based redundancy analysis (db-RDA). The db-RDA that allows non-Euclidean dissimilarity indices (e.g. Bray-Curtis dissimilarity index and Manhattan index) is an extension of the classical redundancy analysis^45^. We selected Bray-Curtis dissimilarity index in this study, as it is less sensitive to the number of species and species absence^46^.

#### Variance partitioning

To further compare the relative effects of different groups of factors (i.e. contemporary climates, historical climate changes and habitat heterogeneity) in shaping species diversity pattern and species composition, we conducted variance partitioning based on partial regressions and partial db-RDA respectively. Using partial regressions, we separated the pure effects of each group of predictors from the combined effects that cannot be ascribe to only one group of predictor due to spatial multicollinearity as follows: (a) pure contemporary climate effects; (b) pure historical climate change effects; (c) pure habitat heterogeneity effects; (d) combined effects of contemporary climate and historical climate changes; (e) combined effects of historical climate change and habitat heterogeneity; (f) combined effects of contemporary climate and habitat heterogeneity; (g) combined effects of contemporary climate, historical climate changes and habitat heterogeneity. As the four frequently-used energy-related variables (i.e. MAT, MTCQ, MTDQ and PET) and four water related variables (i.e. MAP, MPDQ, RAIN and AET) are strongly correlated with each other, we select only one variable from each group to represent contemporary climate in the subsequent analyses to eliminate the influences of multicollinearity on regression analysis. We examined all the 16 possible combinations of one energy-related variable and one water-related variable into regressions/db-RDA and only selected the combinations with the highest adjusted R^2^.

## Results

### Patterns of *Salix* species diversity in eastern Asia

There were 313, 308 and 174 *Salix* species in the whole eastern Asia, mountains and lowlands, respectively. The average *Salix* diversity within grid cells in the whole region, mountains and lowlands were 16 (0–96), 19 (0–96) and 12 (0–40), respectively. The area with the highest species diversity were located in the eastern Tibetan Plateau, Hengduan Montains and its adjacent regions (even extending northward to Shaanxi and Gansu provinces), Northwest of Xinjiang (Tianshan mountain region), northwest of Mongolia, Stanovoy Range and Northeast China (Fig. 1a). The *Salix* diversity in mountains was generally higher than in lowlands (Fig. 1a and b). Except for northeastern China, most of the diversity centers of *Salix* were located in mountains (Fig. 1a and b). In contrast, the *Salix* diversity was relatively low in the western Tibetan Plateau, eastern and southern China and Turan Plain (Fig. 1a).

### Environmental determinants of *Salix* diversity

The direction of the effects of environmental variables (i.e. positive *vs*. negative effects) on *Salix* diversity was consistent across different regions (Table 1). The species diversity decreased with energy-related variables, indicating that *Salix* diversity is higher in colder than warmer regions (Table 1). In contrast, *Salix* diversity was positively correlated with water availability in all three regions (Table 1). MAT anomaly and MAP anomaly were positively correlated with *Salix* diversity (Table 1), indicating that regions where climate were colder and drier during the LGM than today tended to have more *Salix* species than other places. The two variables of topographic heterogeneity (i.e. mean slope and elevational range) were significantly positively correlated with *Salix* diversity.

However, the primary determinants of *Salix* diversity varied in different regions. Mean slope was the strongest single predictor for *Salix* diversity in the whole region and in mountains, accounting for 22.7% and 19.3% of the variance in species diversity respectively. In contrast, MTDQ was the strongest single predictor for species diversity in lowlands and explained 44.4% of species diversity variation. It is noteworthy that the R^2^ of MAP, historical climate change variables and habitat heterogeneity variables were all below 20% in mountains and lowlands (Table 1).

**Figure 2:**
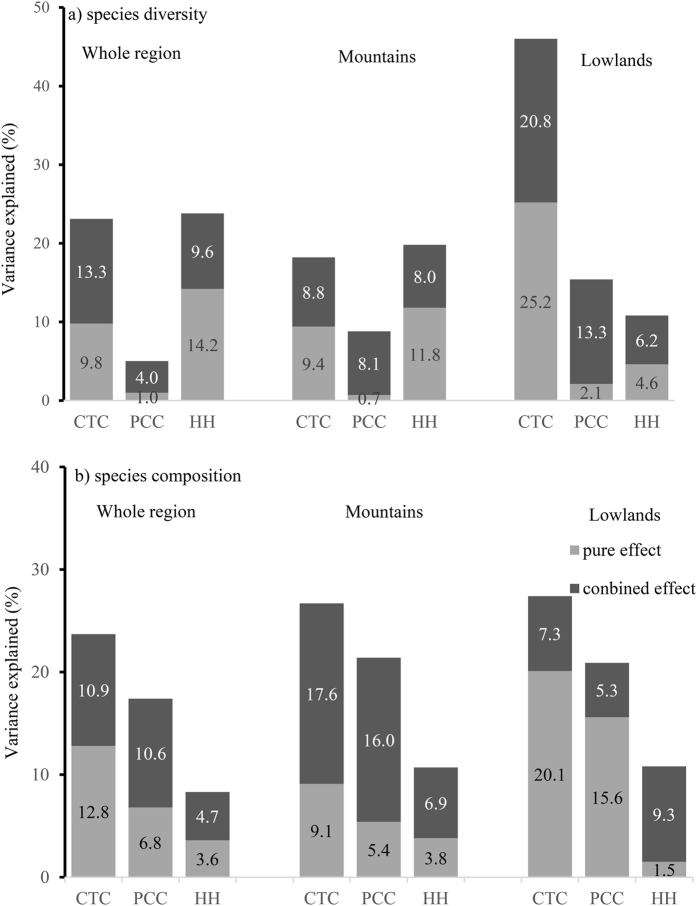
The pure and combined effect of contemporary climate (CTC), historical climate changes (PCC) and habitat heterogeneity (HH) in shaping *Salix* species diversity and species composition in the whole region, maintains and lowlands of eastern Asia. 1) Species diversity; (**b**) species composition. The gray bars indicate the pure effect and black ones indicate the combined effect.

The variance partitioning analysis indicated that the pure effects of habitat heterogeneity accounted for 14.2% of the variance of *Salix* diversity in the whole eastern Asia, while the pure effects of historical climate change accounted for only 1.0%. The pure effects of contemporary climate accounted for 9.8% of species variance of *Salix* diversity patterns (Figs 2a and S4). The relative influences of contemporary climate, historical climate changes and habitat heterogeneity on species diversity were different in mountain and lowland. In mountains, the contemporary climate explained 18.2% of *Salix* species diversity variance. In lowlands, the exploratory power of contemporary climate was much higher (46.0%) (Figs 2a and S4).

### Environmental determinants of *Salix* species composition

**Figure 3:**
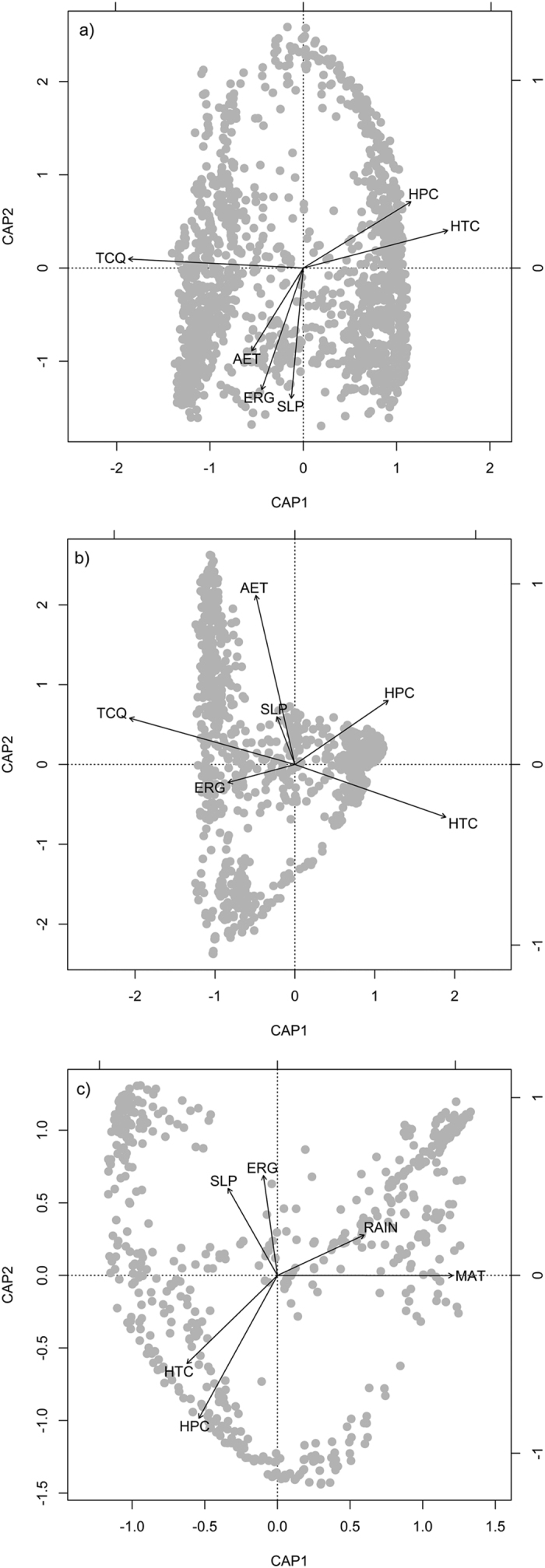
Biplots of distance-based redundancy analysis showing the relationships between *Salix* species composition and environmental factors at the spatial resolution of 100 × 100 km in eastern Asia. (**a**) The whole region; (**b**) mountains; (**c**) lowlands. MAT, mean annual temperature; TCQ, mean temperature of coldest quarter; AET, actual annual evapotranspiration; RAIN, total precipitation of the months with mean monthly temperature >0 °C; HTC, anomaly of temperature between the Last Glacial Maximum and the present; HPC, anomaly of precipitation between the Last Glacial Maximum and the present; SLP, mean slope; ERG, elevation range.

Variables of habitat heterogeneity were always less important than historical climate changes and historical climate changes were always less important than contemporary climate in explaining *Salix* species composition in the whole region, mountains and lowlands. The habitat heterogeneity only explained ca. 10% variance in species composition (Figs 2b and S5). The variance partitioning analysis showed that contemporary climate accounted for about 9.1–20.1% of the variance in *Salix* species composition when the effects of historical climate changes and habitat heterogeneity were controlled for, in the whole region, mountains and lowlands (Fig. 2b). Historical climate changes accounted for 15.6% of species variance in lowlands (Figs 2b and S5) after other factors were controlled for. The first db-RDA axis accounted for about 20.0% variance in species composition (Table 2). Overall, the first db-RDA axis was most strongly correlated with MAT/MTCQ, MAT anomaly and MAP anomaly gradients (Table 2 and Fig. 3a,b,c and d).

## Discussions

In this study, we found that mountains generally harbor more *Salix* species than lowlands. This is consistent with previous reports that regard mountains as species diversity centers of different groups^25^,^47^,^48^. Previous studies have shown that habitat heterogeneity usually contributes less to large-scale species diversity patterns than climate^5^,^47^,^49^. However, we found that contemporary climate is the primary determinants of *Salix* diversity pattern only in lowlands, while mean slope is the primary predictor of *Salix* species diversity in the whole eastern Asia. This is consistent with the finding for birds in Andes^24^. The sites with higher mean slope may imply more riparian habitats that are ideally suitable for *Salix* species. It may also indicate more frequent disturbance events such as landslide and mountain torrents, which creates new space for pioneer species like *Salix* to colonize. Habitat heterogeneity influences *Salix* species distribution probably through the control of the number of available niches in local sites^24^.

More interestingly, we found that contemporary climate accounted for much more variance in *Salix* species composition than habitat heterogeneity. These results suggest that contemporary climate may primarily act as environment filtering forces in shaping individual *Salix* distributions and influence species composition by filtering species according to their different climate preferences^50^.

We obtained negative *Salix* diversity-temperature relationships in this study. Especially in lowland region, MTDQ was the strongest single predictor of *Salix* diversity pattern. Similarly to our results, previous studies have also found that *Salix* diversity in Europe^51^ and Fenoscandia^52^ increases with latitude and is negatively correlated with mean annual temperature^51^. Our results together with previous findings indicate a globally consistent relationship between *Salix* species diversity and temperature and suggest that the mechanisms underlying *Salix* diversity may be the same across different continents.

Given the temperate origination of *Salix*^34^,^53^, such negative diversity-temperature relationship provides support for niche conservatism hypothesis other than ambient energy hypothesis. The contemporary climate may not influence species diversity via the control of resource availability in a region, as suggested by ambient energy hypothesis^5^. In contrast, this is in accordance with the predictions of the niche conservatism hypothesis, which states that the species diversity of a clade decreases with the departure of climate conditions from its ancestral climatic niche^8^,^9^,^10^. Fossil evidence from the late Cretaceous to early Tertiary in northeast China suggest that *Salix* originated in a temperate-like climate conditions^33^,^53^. With regard to temperate origination, *Salix*, therefore, evolved strong cold tolerances^29^,^54^,^55^. Previous study showed that *Salix* twigs could survive freezing temperatures as low as −50 °C and this winter hardiness has been reported even for *Salix* growing in tropical and subtropical regions^54^,^56^. As a clade with temperate-origination, *Salix* species require a period of chilling to de-acclimatize and break bud. In warmer subtropical and tropical regions, many *Salix* species may, therefore, not be able to leaf out, produce flowers or regenerate^28^,^57^. In other words, *Salix* species are less suitable to thrive in low latitude where the climate is much warmer. This could explain why *Salix* species are widely distributed in temperate region of Northern Hemisphere and only mountainous regions of subtropical and tropical regions where climate is much cooler.

However, the high species diversity in Himalaya-Hengduan Mountains seem difficult to be simply explained by niche conservatism hypothesis or by a great number of available ecological niches in this region (i.e. habitat heterogeneity hypothesis) alone. Other variables related with historical and evolutionary processes were not fully considered in this analysis and may significantly contribute to high *Salix* species diversity. Among different historical and evolutionary processes, the uplift of the Qinghai-Tibetan Plateau may have played an important role in promoting rapid diversification of *Salix* species in the Himalaya-Hengduan Mountains and hence making this area as one of the *Salix* diversity centers in the world. Enhanced speciation rate in the Himalaya-Hengduan Mountains have also been found for many other taxonomic groups (e.g. *Larix*^58^; *Rhododendron* subgenus Hymenanthes^59^). These findings together with our results suggest that the Himalaya-Hengduan Mountains may have acted as species cradle not only for *Salix*, but also for many other groups. Future studies based on molecular phylogenies are needed to further reveal the changes in diversification of *Salix* through time in the Himalaya-Hengduan Mountains.

Our analysis indicated that MAT anomaly is weakly positively related with *Salix* diversity. These results do not follow the predictions of the history climate stability hypothesis which states that strong historical climate changes reduce the current species diversity^15^,^16^. We hypothesize that *Salix* with strong freezing tolerance may have found suitable habitats in northern regions during the LGM where the historical climate changes were much stronger than the southern regions. Previous studies^18^,^60^,^61^ have shown that many boreal species were also distributed in the central and eastern Europe including Russian Plain during the LGM. *Salix* produces a large number of tiny and light seeds. The seeds are enveloped by soft cottony hairs in a ring structure which facilitate long-distance dispersal via wind or by water current^62^. Strong dispersal ability may have facilitated re-colonization of *Salix* species since the LGM. This hypothesis is consistent with the previous findings on European plant distribution. Specifically, studies on the European plants found that species with strong dispersal ability recolonized most climatically suitable areas in northern Europe after the LGM, while those with weak dispersal ability retained in the southern Europe^63^.

We also detected signals of historical climate changes on *Salix* species composition in eastern Asia. Previous studies showed that species compositions could keep track with climate changes^20^,^21^. Species tolerance to climate changes and the migration rate varies among species and the ability of species to track with climate changes are different^21^. Strong historical climate changes may have possibly made an unsuitable condition for some *Salix* species that have weak tolerance to climate changes. In addition, *Salix* species are pioneer species^32^, which quickly occupies new open habitat. Strong historical climate changes in the high latitude may have create vacant habitats for pioneer *Salix* to colonize and persist and hence shifting the species compositions of *Salix*. Previous studies based on pollen evidences pointed out that *Salix* species were widely distributed in the Arctic regions during warmer post-LGM periods^64^,^65^. In the recent decades, the Arctic regions have experienced rapid climate warming than other regions. The warming climate could lengthen the growing seasons of shrubs and increase the nutrients availability via enhanced microbial activity^66^. *Salix* is one of the major groups in pan-Arctic vegetation. The recent and future climate warming could remarkably influence the distribution and composition of *Salix* species, thereby bringing about great changes in the vegetation structure and function in pan-Arctic regions. However, the number of relevant studies in this direction are still quite few^67^.

## Summary

In this study, we found evidence for niche conservatism, historical climate changes and habitat heterogeneity to explain the diversity and species composition of *Salix* species in eastern Asia. However, other important factors such as disturbance history, diversification rate, soil moisture regime of local habitat^68^ and herbivores^69^ were not included in the analyses due to problems in obtaining data. In addition, *Salix* species are typical pioneer plants with strong dispersal and colonization abilities, and are able to fill a newly created or empty habitat. Therefore, many *Salix* species could easily cover a wide elevational and latitudinal range^29^,^30^,^31^,^32^. Particularly because of this tendency of *Salix*, variation in species distribution could not be easily captured by a single group of environmental factor in this study.

## Additional Information

**How to cite this article**: Wang, Q. *et al*. Historical factors shaped species diversity and composition of *Salix* in eastern Asia. *Sci. Rep.* **7**, 42038; doi: 10.1038/srep42038 (2017).

**Publisher's note:** Springer Nature remains neutral with regard to jurisdictional claims in published maps and institutional affiliations.

## Supplementary Material

Supplementary Information

## Figures and Tables

**Table 1:** Explanatory power (R^2^) of the predictors for *Salix* species diversity with a spatial resolution of 100 × 100 km evaluated by general linear models in the whole study region, mountains and lowlands seperately.

	Whole region	Mountains	Lowlands
**Modern climate**			
MAT	8.2% (−)*	2.5% (−)	31.9% (−)**
MTCQ	7.4% (−)	2.4% (−)	33.8% (−)***
MTDQ	15.3% (−)**	6.3% (−)*	44.4% (−)***
PET	7.2% (−)*	2.8% (−)	32.5% (−)***
MAP	1.0% (+)	3.1% (+)	0.5% (+)
RAIN	1.0% (+)	2.5% (+)	2.4% (+)
PDQ	1.0% (+)	0.2% (+)	0.2% (+)
AET	1.8% (+)	4.8% (+)***	0.2% (+)
**Historical Climate change**			
MAT Anomaly	0.9% (+)	0.1% (+)	15.5% (+)**
MAP Anomaly	5.0% (+)*	8.4% (+)***	6.8% (+)
**Habitat heterogeneity**			
Mean slope	22.7% (+)***	19.3% (+)***	10.3% (+)
Elevation range	15.6% (+)***	9.7% (+)***	6.1% (+)

Modified-T tests were used to test the significance. *P < 0.05, **P < 0.01, ***P < 0.001.

**Table 2:** Biplot scores for constraining variables for the first axes in distance-base redundancy analyses.

	The whole region	Mountains	lowlands
Total variance explained by the first canonical axes	20.0%	23.1%	21.0%
MAT	—	—	0.98
MTCQ	−0.93	−0.91	—
AET	−0.27	−0.22	—
RAIN	—	—	0.49
MAT anomaly	0.77	0.83	−0.51
MAP anomaly	0.57	0.51	−0.44
Mean slope	−0.06	−0.10	−0.27
Elevation range	−0.22	−0.37	−0.08
