# Supplementary material for: Historical factors shaped species diversity and composition of Salix in eastern Asia
Source: Sci Rep. 2017 Feb 8;7:42038. doi: 10.1038/srep42038 (PMC5296738; doi:10.1038/srep42038)
Supplement: Supplementary Information [file srep42038-s1.doc]

**SUPPLEMENTARY INFORMATION**

**Historical factors shaped species diversity and composition of *Salix* in eastern Asia**

Qinggang Wang, Xiangyan Su, Nawal Shrestha,Yunpeng Liu, Siyang Wang, Xiaoting Xu, Zhiheng Wang^*^

Department of Ecology, College of Urban and Environmental Sciences, and Key Laboratory of Earth Surface Processes of Ministry of Education, Peking University, Beijing 100871, China

*Author for correspondence: Zhiheng Wang; Tel: +86 10 62760881; Email: zhiheng.wang@pku.edu.cn

**Table S1:** Explanatory power (R^2^) of the predictors for *Salix* species diversity evaluated by general linear models in the whole study region, mountains and lowlands with a spatial resolution of 50 × 50 km .

**Table S2:** Biplot scores for constraining variables for the first axes in distance-base redundancy analyses with a spatial resolution of 50 × 50 km.

**Table S3:** Mean values and the ranges of six environmental variables (i.e. mean annual temperature MAT, mean annual precipitation MAP, anomaly of temperature and precipitation between present and the last Glacial Maximum MAT Anomaly and MAP Anomaly, mean slope and elevation range) with a spatial resolution of 100 × 100 km used in the analyses.

**Figure S1:** The distribution pattern of *Salix* species diversity in eastern Asia estimated in an equal-area grid of 50×50 km and the topography map of eastern Asia. a) The topography map; b) the distribution pattern of *Salix* diversity. The maps were created in ArcGIS 10 (http://www.esri.com/software/arcgis). The topography map in the figures was generated based on Global 30 Arc-second Elevation (GTOPO30) of U.S. Geological Survey (https://lta.cr.usgs.gov/GTOPO30).

**Figure S2**: The pure and combined effect of contemporary climate (CTC), historical climate changes (PCC) and habitat heterogeneity (HH) in shaping *Salix* species diversity and species composition with a spatial resolution of 50 × 50 km in whole region, maintains and lowlands of eastern Asia.

**Figure S3:** Distance-based redundancy analysis biplot showed the relationship between *Salix* species composition and environment factors with a spatial resolution of 50 × 50 km in eastern Asia.

**Figure S4:** The pure and combined effect of contemporary climate (CTC), past climate change (PCC) and habitat heterogeneity (HH) in shaping *Salix* species diversity with a spatial resolution of 100 × 100 km in whole regions, maintain regions and lowland regions of eastern Asia.

**Figure** **S5:** The pure and combined effect of contemporary climate (CTC), past climate change (PCC) and habitat heterogeneity (HH) in shaping Salix species composition with a spatial resolution of 100 × 100 km in whole regions, maintain regions and lowland regions of eastern Asia

**Table S1.** Explanatory power of the predictors for *Salix* species diversity evaluated by general linear models with a spatial resolution of 50 × 50 km in the whole study region, mountains and lowlands. Modified-T tests were used to test the significance. * P<0.05, ** P<0.01, ***P<0.001.

|  | Whole region | Mountains | Lowlands |
| --- | --- | --- | --- |
| **Modern climate** |  |  |  |
| MAT | 12.4% (-)** | 4.9% (-) | 32.8% (-)** |
| MTCQ | 12.0% (-)* | 5.8% (-) | 34.5% (-)*** |
| MTDQ | 12.0% (-)* | 5.6% (-) | 34.5% (-)*** |
| PET | 10.9% (-)* | 4.8% (-) | 32.4% (-)*** |
| MAP | 0.1% (+) | 0.4% (+) | 0.3% (+) |
| RAIN | 0.0% (+) | 0.1% (+) | 1.8% (+) |
| PDQ | 3.8% (+)*** | 3.1% (+) | 1.6% (+)* |
| AET | 0.8% (+) | 1.5% (+) | 0.0% (+) |
| **Historical Climate change** |  |  |  |
| MAT Anomaly | 2.4% (+) | 1.1% (+) | 15.7% (+)*** |
| MAP Anomaly | 9.4% (+)*** | 11.2% (+)*** | 12.3% (+)* |
| **Habitat heterogeneity** |  |  |  |
| Mean slope | 18.8% (+)*** | 11.5% (+)*** | 11.3% (+) |
| Elevation range | 14.3% (+)*** | 5.5% (+)** | 7.6% (+) |

**Table S2.** Biplot scores for constraining variables for the first axes in distance-base redundancy analyses with a spatial resolution of 50 × 50 km

|  | The Whole region | Mountains | lowlands |
| --- | --- | --- | --- |
| Total variance explained by the first canonical axes | 18.9% | 22.3% | 19.2% |
| MAT | - | - | 0.98 |
| MTCQ | -0.93 | -0.91 | - |
| AET | -0.26 | -0.26 | - |
| RAIN | - | - | 0.39 |
| MAT anomaly | 0.77 | 0.85 | -0.51 |
| MAP anomaly | 0.59 | 0.51 | -0.49 |
| Mean slope | 0.02 | -0.07 | -0.37 |
| Elevation range | -0.12 | -0.31 | -0.19 |

**Table S3.** Mean values and the ranges of six environmental variables (i.e. mean annual temperature MAT, mean annual precipitation MAP, anomaly of temperature and precipitation between present and the last Glacial Maximum MAT Anomaly and MAP Anomaly, mean slope and elevation range) with a spatial resolution of 100 × 100 km used in the analyses.

|  | All region | | Mountains | Lowlands |  |
| --- | --- | --- | --- | --- | --- |
| MAT (°C) | -0.6 [-20.6, 24.1] | | -0.74 [-20.6, 21.2] | -0.2 [-17.3, 24.1] |  |
| MTCQ (°C) | -17.4 [-46.9, 18.7] | | -17.4 [-46.9,16.0] | -17.6 [-43.0, 18.7] |  |
| MTDQ(°C) | -12.8 [13.7, 15.1] | | -13.5 [-43.0,26.9] | -11.6[-38.3, 28.6] |  |
| PET | 709[137, 1505] | | 726[137,1506] | 681[154, 1451] |  |
| MAP (mm) | 457 [17, 2778] | | 461 [17, 2778] | 451 [19, 2021] |  |
| RAIN (mm) | 367 [0, 2778] | | 381[0,2778] | 3344[18, 2021] |  |
| PDQ (mm) | 37 [0, 412] | | 31 [0,412] | 46.5[0, 198] |  |
| AET | 360 [19,1182] | | 365 [19,1155] | 353 [21, 1182] |  |
| MAT Anomaly (°C) | | 8.5 [2.3, 18.6] | 8.2 [1, 17.8] | 9.1 [0.2, 018.5] |  |
| MAP Anomaly (mm) | | 140[1,626] | 132 [1, 626] | -153[4,391] | |
| Mean slope (°) | 2.5 [0, 16.9] | | 3.5 [0.1, 16.9] | 0.7 [0. 02, 4.8] |  |
| Elevation range (m) | 1154 [11, 6183] | | 1582[3016183] | 435 [11, 3186] |  |


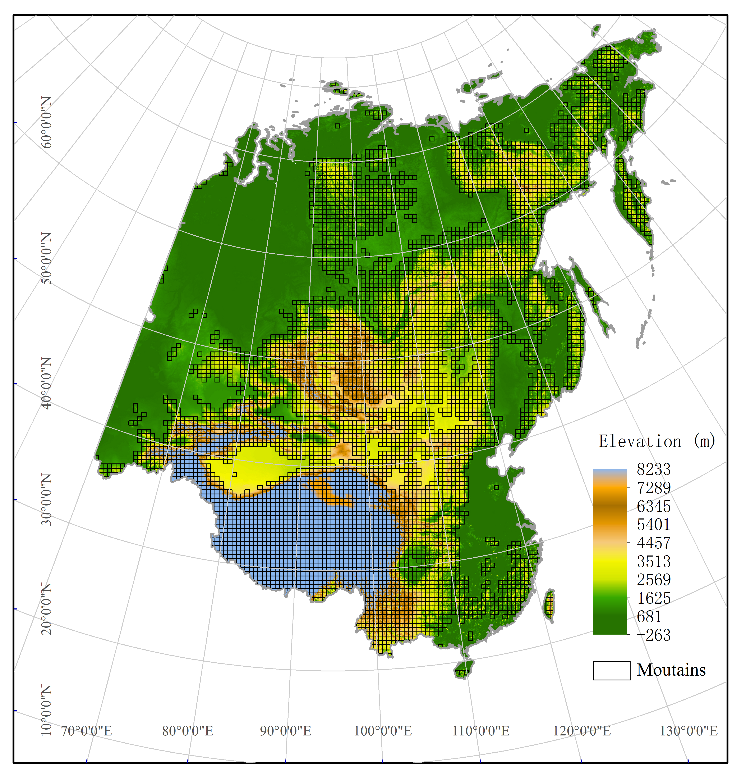


a)


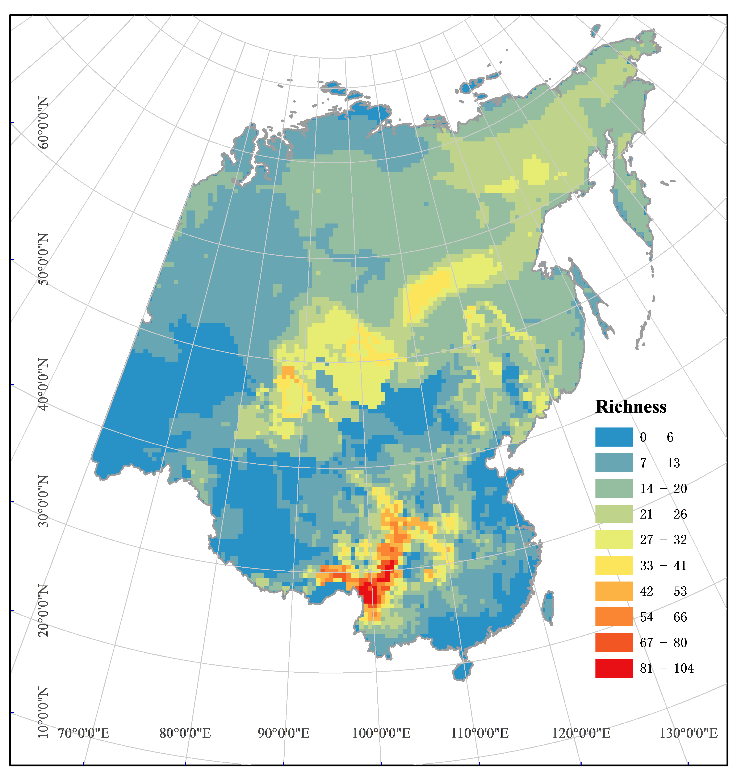


b)

**Figure S1**. The distribution pattern of *Salix* species diversity in eastern Asia estimated in an equal-area grid of 50×50 km and the topography map of eastern Asia. a) The topography map; b) the distribution pattern of *Salix* diversity. The maps were created in ArcGIS 10 (<http://www.esri.com/software/arcgis>). The topography map in the figures was generated based on Global 30 Arc-second Elevation (GTOPO30) of U.S. Geological Survey (https://lta.cr.usgs.gov/GTOPO30).


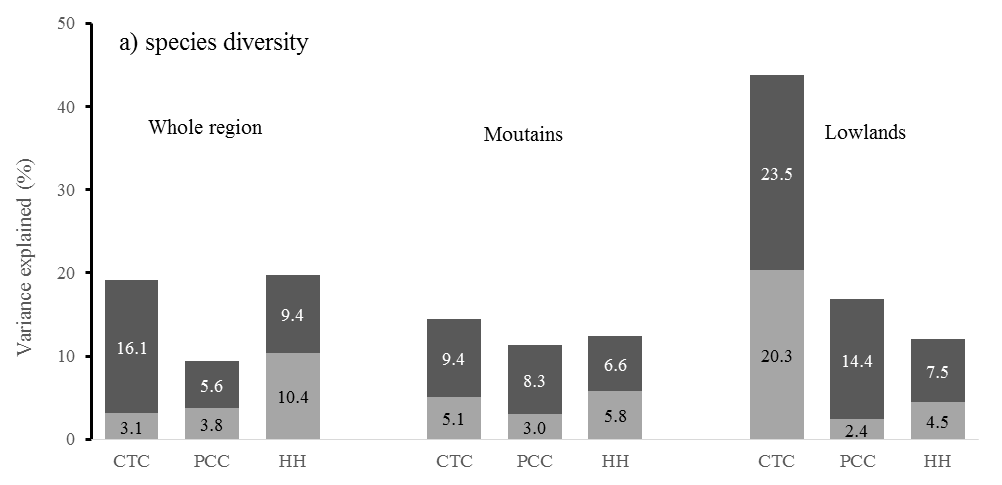


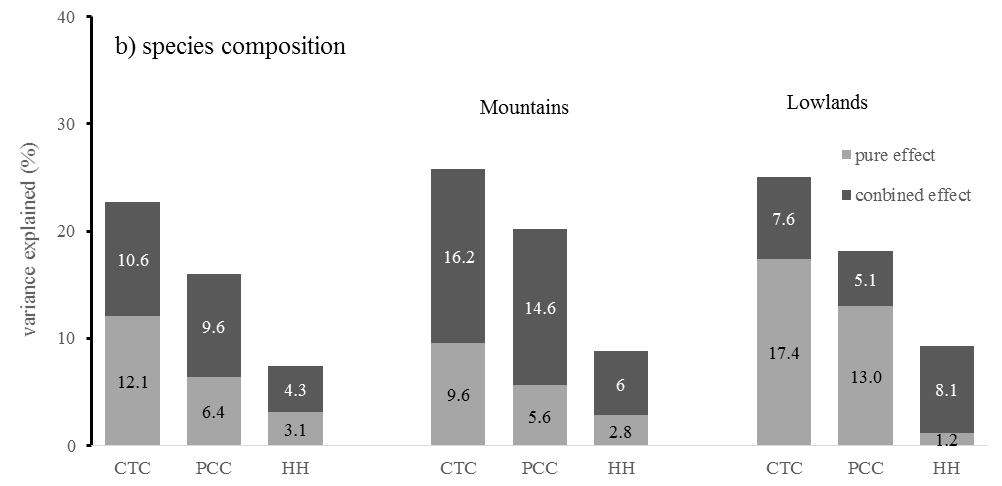


Whole region

Figure S2. The pure and combined effect of contemporary climate (CTC), historical climate changes (PCC) and habitat heterogeneity (HH) in shaping *Salix* species diversity and species composition with a spatial resolution of 50 × 50 km in whole region, maintains and lowlands of eastern Asia. 1) Species diversity; b) species composition. The gray bar indicates the pure effect and black indicates the combined effect.


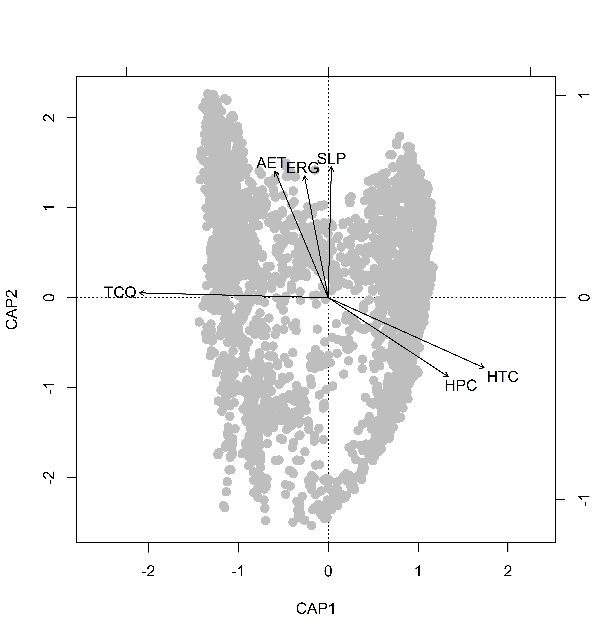

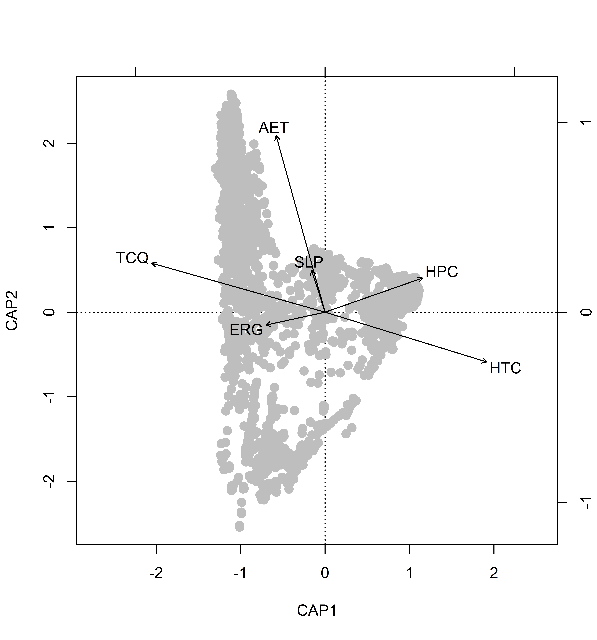

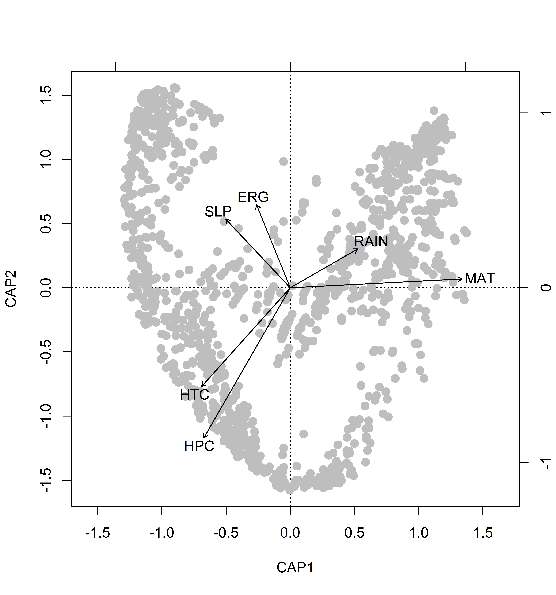


a)

b)

c)

**Figures S3.** Biplots of distance-based redundancy analysis showing the relationship between *Salix* species composition and environment factors with a spatial resolution of 50 × 50 km in whole region, mountains and lowlands of eastern Asia. a) Whole regions; b) Mountains; c) Lowlands. MAT, mean annual temperature; TCQ, mean temperature of coldest quarter ; AET, actual annual evapotranspiration; RAIN, total precipitation of the months with mean monthly temperature > 0 ^o^C; HTC, anomaly of temperature between the Last Glacial Maximum and the present; HPC, anomaly of precipitation between the Last Glacial Maximum and the present; SLP, mean slope; ERG, elevation range.


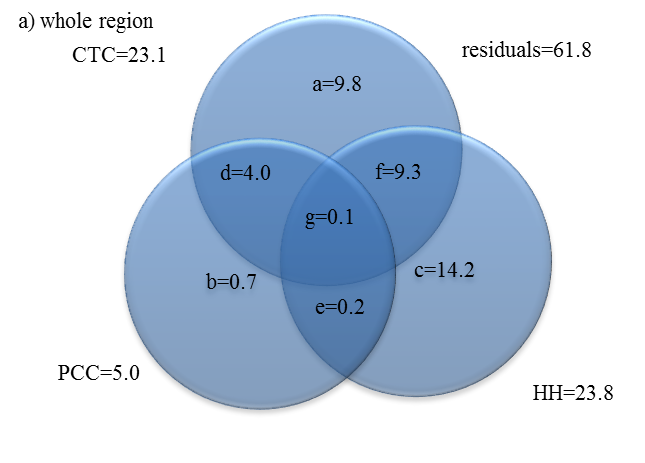


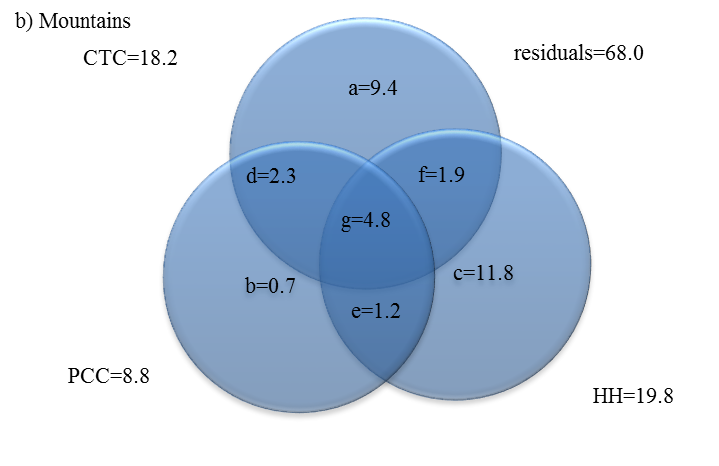


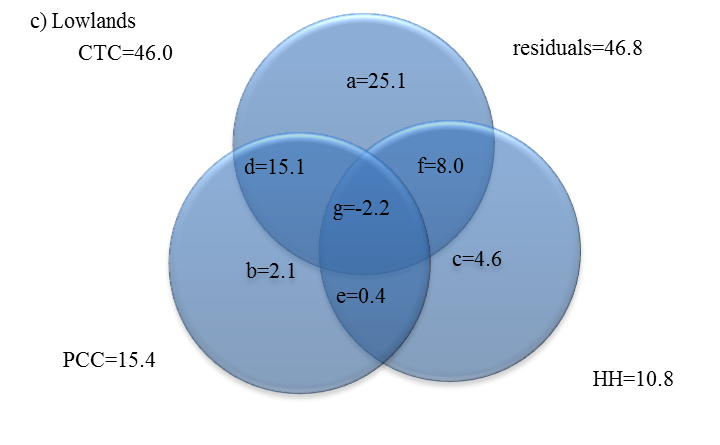


**Figure S4.** The pure and combined effect of contemporary climate (CTC), past climate change (PCC) and habitat heterogeneity (HH) in shaping *Salix* species diversity with a spatial resolution of 100 × 100 km in whole region, maintains and lowlands of eastern Asia. a) All regions; b) Mountains; c) Lowlands.


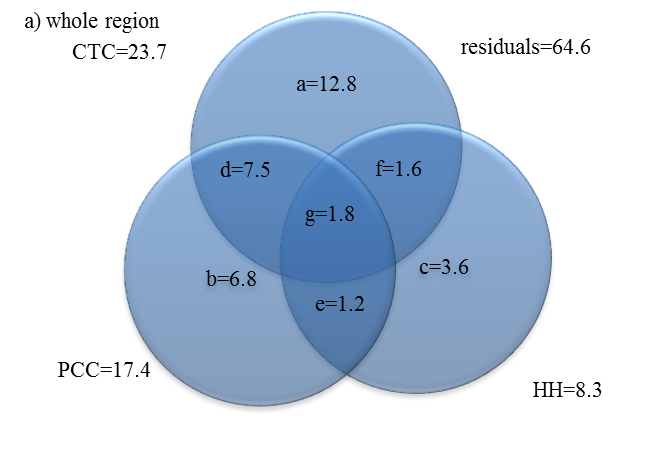


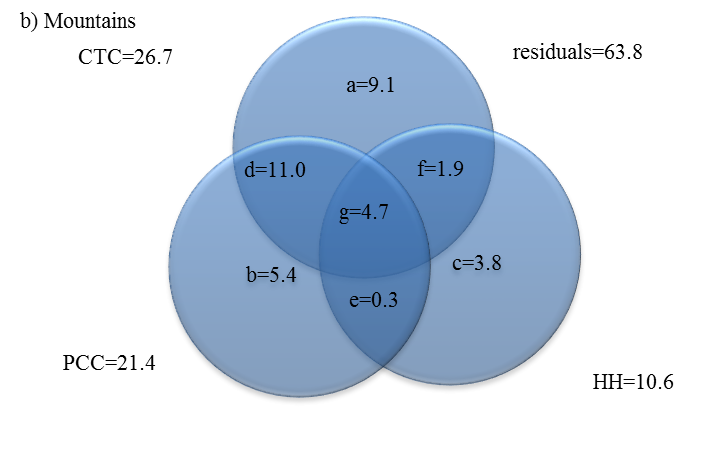


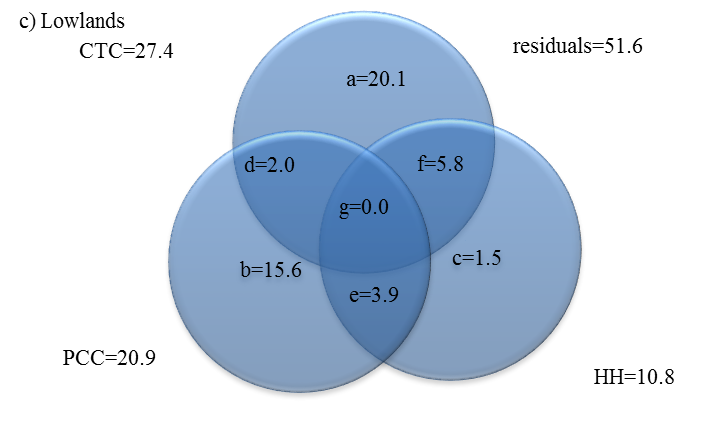


**Figure S5.** The pure and combined effect of contemporary climate (CTC), past climate change (PCC) and habitat heterogeneity (HH) in shaping *Salix* species composition with a spatial resolution of 100 × 100 km in whole region, maintains and lowlands of eastern Asia. A) All regions; b) Mountain regions; c) Lowland regions.
